# Innate epithelial and functional differences in airway epithelium of children with acute wheeze

**DOI:** 10.3389/fcell.2025.1606915

**Published:** 2025-07-28

**Authors:** Kevin Looi, Thomas Iosifidis, Saraya Harrison, Stephen M. Stick, Peter LeSouef, Ingrid A. Laing, Anthony Kicic

**Affiliations:** ^1^Wal-yan Respiratory Research Centre, The Kids Research Institute Australia, The University of Western Australia, Nedlands, WA, Australia; ^2^School of Population Health, Curtin University, Bentley, WA, Australia; ^3^Centre for Child Health Research, The University of Western Australia, Nedlands, WA, Australia; ^4^Centre for Cell Therapy and Regenerative Medicine, School of Medicine and Pharmacology, The University of Western Australia and Harry Perkins of Institute of Medical Research, Nedlands, WA, Australia; ^5^School of Medical, Molecular and Forensic Sciences, Murdoch University, Perth, WA, Australia; ^6^Department of Respiratory and Sleep Medicine, Perth Children’s Hospital, Nedlands, WA, Australia

**Keywords:** acute wheeze, airway epithelium, barrier integrity, cell culture, conditional reprogramming

## Abstract

**Background:**

Early childhood wheeze is a major risk factor for asthma. However, not all children who wheeze will develop the disease. The airway epithelium has been shown to be involved in asthma pathogenesis. Despite this, the airway epithelium of children with acute wheeze remains poorly characterized.

**Methods:**

Upper airway epithelial cells (AEC) from children with acute wheeze and non-wheeze controls were cultured and expanded. Markers of epithelial lineage (Cytokeratin (KRT)-5, −19) and vimentin were assessed via qPCR and immunocytochemistry. Inflammatory cytokines (Interleukin (IL)-1β, −6, and −8) were measured using ELISA. Tight junction (TJ) protein expression and barrier integrity were determined via In-Cell Western and paracellular permeability assays, respectively.

**Results:**

Upper AECs from children with acute wheeze had significantly higher *KRT19* and lower *vimentin* gene expression compared to non-wheeze controls but similar *KRT5* levels. Similar staining intensities of KRT5 and KRT19 proteins were observed in both cohorts. IL-6 and IL-8 levels were not significantly different, but IL-1β was increased in cultures from children with acute wheeze compared to controls. Tight junction protein expression of claudin-1, occludin and ZO-1 were significantly lower in acute wheeze cohorts, concomitant with increased paracellular permeability.

**Conclusion:**

Airway epithelium of children experiencing acute wheeze appears abnormal, primarily with compromised epithelial barrier integrity.

## 1 Introduction

The respiratory airways, commencing from the nasal cavity and extending to the distal sites to include the respiratory bronchioles and alveoli, are the initial sites where interaction with the external environment first occurs. Although the primary role of the respiratory epithelium is to facilitate efficient gas exchange, it also provides a pivotal role in maintaining respiratory homeostasis through constant immunological surveillance and the organization of host defenses (Holgate, 2011; Gohy et al., 2020). In a healthy state, the epithelium responds to external stimuli by initiating and orchestrating a coordinated and transient inflammatory response to restore barrier integrity. However, we have shown that in susceptible individuals, this epithelial response is dysregulated (Looi et al., 2018; Iosifidis et al., 2020). When this dysregulated epithelium encounters external challenges, frequent exacerbations and consequent disease pathogenesis can often occur. Airway diseases such as asthma is the most prevalent respiratory disease globally, affecting more than 300 million people of all ethnic groups throughout all ages and is the most common chronic airway disease in children (Soriano et al., 2020). Despite the identification of various asthma phenotypes in children, it is still known as an inflammatory disease of the airways, characterized by symptoms such as wheezing, shortness of breath, chest tightness and/or cough which may resolve either spontaneously or following medical intervention (Fanta, 2009; Holgate et al., 2015).

Wheezing has been considered to be an important characteristic of asthma and has been associated with respiratory viral infections in individuals of all ages. For most children, wheezing episodes associated with respiratory viral infections gradually diminish with age but for a sub-set of children, early life wheezing episodes are often a prelude to asthma (Kenmoe et al., 2020). Meta-analysis of wheezing episodes and respiratory viral infections during early life have provided an opportunity to elucidate the potential relations between these episodes and subsequent asthma development (Makrinioti et al., 2022). Past studies investigating asthma have mainly used airway epithelial cells derived from adults with either mild or moderate form of the disease (de Boer et al., 2008; Xiao et al., 2011). However, these are often not the most accurate biological replicate of wheezing conditions. Moreover, despite airway epithelial cells derived from adults presenting with wheeze being a closer representation, these cells are temporally separated from the childhood conditions and may present other confounding factors. Thus, the present limitation to further investigations of childhood wheeze has been the inability to safely obtain airway epithelial cells from children during a wheezing episode.

Studies that have used conditional reprogramming of airway epithelial cells, which involves the co-culturing with irradiated murine fibroblasts as feeder cells, have demonstrated the feasibility of this methodology in extending culture success. These studies showed improved culture expansion rates, with overall increase in cell yields while retaining the epithelial lineage characteristics and disease phenotype over successive expansions (Martinovich et al., 2015; Wolf et al., 2017). Moreover, a seminal study by Kicic et al. demonstrated transcriptional similarities between cells obtained from the upper and lower airways of children and also showed that variations associated with disease characteristics are similarly conserved (Kicic et al., 2020). As there have not been comprehensive studies using epithelial cells derived from children with acute wheeze, this study presents the first detailed examination of the airway epithelium from children with acute wheeze with the aim of testing the hypothesis that there are intrinsic differences between the epithelium of children with acute wheeze and non-wheeze controls. To address this hypothesis, we used conditional re-programming on upper airway epithelial cells derived from children with wheeze and non-wheeze controls to elucidate potential differences in epithelial responses and barrier integrity.

## 2 Materials and methods

### 2.1 Reagents

Bovine serum albumin, fetal bovine serum, bovine hypothalamus acetone power, hydrocortisone, recombinant human epidermal growth factor, epinephrine hydrochloride, fibronectin, rat-tail type I collagen, triiodothyronine, transferrin, trans-retinoic acid, trypsin, and gentamicin were obtained from Sigma-Aldrich (St. Louis, MO, United States), fibronectin (BD, Franklin Lakes, NJ, United States), Y-27632 ROCK inhibitor (Enzo Life Sciences, Farmingdale, NY, United States) and all tissue culture plastic ware were purchased from Corning (Corning, NY, United States). ReagentPack™ Subculture Reagents, Bronchial epithelial basal medium (BEBM™) and bronchial epithelial cell growth medium (BEGM™) were purchased from LONZA (Melbourne, VIC, Australia). RT-PCR and qPCR reagents were sourced from Thermo Fisher Scientific. Antibodies against claudin-1 (polyclonal), occludin (monoclonal, clone OC-3F10), zonula occludens (ZO)-1 (monoclonal, clone ZO1-1A12), e-cadherin (monoclonal, clone HECD-1), PTEN (monoclonal, clone 2F4C9), EGFR (monoclonal, clone 111.6), KRT5 (monoclonal, clone 2C2), KRT19 (monoclonal, clone A53-B/A2.26 (Ks19.1)) and vimentin (monoclonal, clone V9) were also obtained from Thermo Fisher Scientific. 4′,6-diamidino-2-phenylindole (DAPI) was sourced from Sigma-Aldrich. AlexaFluor 488 (Goat anti-Mouse and Goat anti-Rabbit) were purchased from Life Technologies.

### 2.2 Study participants and sample collection

Primary upper airway epithelial cells (AEC) were used from 2 separate cohorts in this study. The non-wheeze cohort consisted of 13 children with no history of wheeze, that were undergoing elective surgery for non-respiratory related conditions as part of the Western Australia Epithelial Research Program (WAERP). Upper AECs were obtained as previously described (Kicic et al., 2020). These samples were age- and sex-matched to the acute wheeze cohort. For the acute wheeze cohort, upper AECs were obtained via a nasal swab of the inferior turbinate from 13 children presenting to the emergency department at Perth Children’s Hospital with a wheeze related exacerbation and recruited into the Mechanisms of Acute Viral Respiratory Infection in Children (MAVRIC) study. The study was approved by Perth Children’s Hospital, St John of God Hospital, Subiaco and The University of Western Australia’s Human Ethics Committees and written consent was obtained from each participant’s legal guardian after being fully informed about the nature and purpose of the study.

### 2.3 Airway epithelial cell cultures

Upper AECs were co-cultured with irradiated murine embryonic fibroblast (NIH-3T3) feeder cells, in the presence of Rho-associated protein kinase (ROCK) inhibitor Y-27632, on tissue culture-treated plastic flasks pre-coated with extracellular matrix components, fibronectin and type I collagen. When the co-cultures reached approximately 90% confluence, they were passaged by differential trypsinization using a Trypsin/EDTA reagent pack. This was performed to remove the feeder cells from the epithelial culture based on their differential trypsin sensitivity. Growth medium was aspirated and cells rinsed with 1 mL of 1×PBS for 2 min prior to incubation in 1 mL of Trypsin/EDTA at room temperature for 2 min until fibroblasts had rounded up and lifted off. Cells were then rinsed with 1 mL of HEPES-Buffered Saline Solution (HBSS) for 2 min, aspirated off and incubated in 1 mL of Trypsin/EDTA solution at 37°C for 7 min or until epithelial cells had begun to detach from the tissue culture vessel. Trypsinization was halted by addition of 1 mL of Trypsin Neutralizing Solution. Cells were then collected, centrifuged at 500 g for 7 min at 4°C, resuspended in appropriate culture medium and enumerated. Viability was assessed using the trypan blue exclusion method. Upper AECs from both cohorts of children were established and expanded using this conditional reprogramming method, as previously described (Martinovich et al., 2015). This facilitated the expansion and passage of primary upper AECs while retaining key epithelial lineage and disease characteristics. All endpoint experiments were performed in bronchial epithelial growth media (BEGM) supplemented with additives, as previously described (Looi et al., 2018).

### 2.4 Cell proliferation assay

Cell proliferation of both cohorts were assessed using a 3-[4,5-dimethylthiazol-2yl]-5-[3-carboxymethoxyphenyl]-2-[4-sulfophenyl]-2H-tetrazolium inner salt (MTS) assay (Promega; Madison, WI, United States) as previously described (Iosifidis et al., 2020).

### 2.5 RNA extraction and gene expression analysis

Total RNA was extracted using the Ambion PureLink RNA mini kit per manufacturer’s instructions. The mRNA expression of *KRT5, KRT19, VIM* and housekeeping gene, *PPIA*, were determined using specific primers (Table 1). Gene expression was determined via a two-step reverse transcription and real-time qualitative PCR (RT-qPCR) assay. Relative gene expression was calculated, as previously described (Martinovich et al., 2015), using the comparative CT (2^−ΔΔCT^) method. Briefly, for each sample, the cycle threshold (CT) value for the gene of interest was normalized to the CT value of the housekeeping gene (*PPIA*) to generate the delta CT (Δ*CT*) value. To calculate the final relative expression, the Δ*CT* value for each individual samples was then calibrated against the average Δ*CT* of the entire non-wheeze cohort, which served as the endogenous tissue control for this study. The resulting relative quantification values (2^−ΔΔCT^) are presented throughout the results as ‘arbitrary unit (AU)’, where the biological mean of the non-wheeze control group is normalized to 1.

**TABLE 1:** Oligonucleotide primer sequences.

Gene of interest	Primer	Sequence
KRT5	Forward	5′TGGAGATCGCCACTTACCG3′
	Reverse	5′CCAGAGGAAACACTGCTTGTG3′
KRT19	Forward	5′ATAAAAGCCAGGTGAGG3′
	Reverse	5′GCTGTAGGAAGTCATGGCGA3′
VIM	Forward	5′GAGGAGATGCGGGAGCTG3′
	Reverse	5′ATGATGTCCTCGGCGAGGTT3′
PPIA	Forward	5′CCTTGGGCCGCGTCTCCTTT3′
	Reverse	5′CACCACCCTGACACATAAACCCTGG3′

### 2.6 Immunocytochemistry

Upper AECs from both cohorts cytospun onto slides (50 × 10^3^ cells/slide) were stained for epithelial lineage markers: Cytokeratins (KRT)-5, −19 and mesenchymal marker: Vimentin (VIM) as previously described (Martinovich et al., 2015).

### 2.7 Cytokine assay

Cytokine concentrations of interleukin (IL)-1β, −6 and −8 were assessed in collected supernatants using commercial ELISA kits as previously described (Kicic et al., 2016). Briefly, each kit was a solid phase sandwich ELISA using monoclonal antibodies specific to the target cytokine. Biotinylated secondary antibodies were used in the detection of the immobilized capture antibodies and streptavidin-peroxidase used as the detection agent. Standard curves were generated using serial dilutions and sample concentrations normalized to protein concentrations.

### 2.8 In cell Western^™^ assay

Protein expression of TJ and AJ proteins, claudin-1, occludin, zonula occludens (ZO)-1 and e-cadherin, EGFR and the tumor suppressor, PTEN, were determined using specific antibodies and quantified as previously described (Looi et al., 2018). Protein expression was quantified using a two NIR laser imaging system for the excitation of NIR fluorescent dye conjugated-secondary antibodies bound onto the target proteins. Quantified protein expression was normalized to cell number stained with cellular nuclear stains (DRAQ5™ and Sapphire700™).

### 2.9 Paracellular permeability

Upper airway epithelial cells from children with and without acute wheeze were plated at a seeding density of 1.5 × 10^6^ cells/cm^2^ onto pre-coated Transwell 24-well inserts (0.4 µm PET, CLS3470, Corning). When a confluent monolayer was achieved, culture media was removed and paracellular permeability across the monolayer culture was assessed using FITC-dextran of two sizes (4 and 20 kDa). Apparent permeability (P_*app*_) was calculated following the general equation: P_*app*_ = (*dQ/dt) × (1/AC*_*0*_ *),* where dQ/dt is the steady-state flux, A is the surface area of the membrane and C_0_ is the initial concentration in the donor compartment as previously described (Looi et al., 2018).

### 2.10 Statistics

Experiments were performed in at least technical duplicates with biological replicates indicated in each figure legend and values are reported as mean ± standard deviation where appropriate. Normality of data was determined using D’Agostino & Pearson normality test. Unpaired t-test was used for parametric data, and Mann-Whitney statistical test for non-parametric data. Correlation or lack thereof was assessed through Spearman non-parametric correlation test. All p values less than 0.05 were considered significant. GraphPad Prism 10 software package was used for performing statistical analyses and graphical representation of data.

## 3 Results

### 3.1 Successful culture establishment of AECs from children with acute wheeze

Study cohorts consisted of upper AEC collected from 13 non-wheezing children (average age 2.7 ± 0.3 years; 6 male), and 13 children experiencing an acute wheeze episode resulting in hospital presentation (average age 3.1 ± 1.3 years; 7 male). There was no significant difference between the composition of the cohorts in terms of age or sex (Table 2).

**TABLE 2:** Demographics of non-wheeze and acute wheeze cohort.

Cohort parameters	Non-wheeze	Acute wheeze	p-value
Number of participants	13	13	-
Mean age of participants (years)	3 (2.1–8.5)	4.4 (1.6–10)	>0.05
Male (%)	46	54	>0.05
Wheeze ever (%)	23	100	<0.001
Atopy (%)	15	69	<0.05
Parental asthma (%)	46	31	>0.05

**FIGURE 1:**
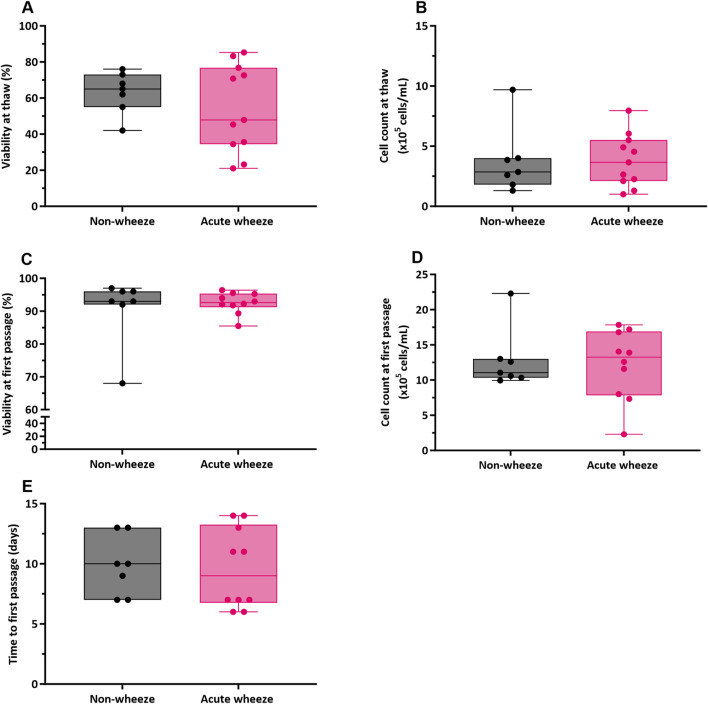
Airway epithelial cell viability, yields and time to first passage between non-wheeze and acute wheeze cell cultures. **(A)** Cell viability at thaw between acute wheeze (n = 11) and non-wheeze (n = 7) cultures were not significantly different, with mean cell viabilities of 54% ± 24% and 63% ± 12% respectively. **(B)** Live cell yield, as assessed via trypan-blue exclusion were also non-significantly different at thaw between acute wheeze (3.81 ± 2.18 × 10^5^ cells/mL; n = 11) and non-wheeze (3.73 ± 1.25 × 10^5^ cells/mL; n = 7) cultures. **(C)** At confluence, cultures were passaged and cell viability obtained were 93% ± 3% and 91% ± 11% for the acute wheeze (n = 10) and non-wheeze (n = 7) cultures respectively. **(D)** Live cell yield at the first passage, similarly assessed via trypan blue exclusion, were not significantly different between acute wheeze (12.17 ± 4.99 × 10^5^ cells/mL; n = 10) and non-wheeze (11.26 ± 1.25 × 10^5^ cells/mL; n = 7) cultures. **(E)** Both acute wheeze (n = 10) and non-wheeze (n = 7) cohorts reached confluence and ready for the first passage at 9.6 ± 3.3 days and 9.86 ± 2.5 days respectively. 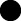
Non-wheeze, 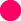
Acute wheeze.

**FIGURE 2:**
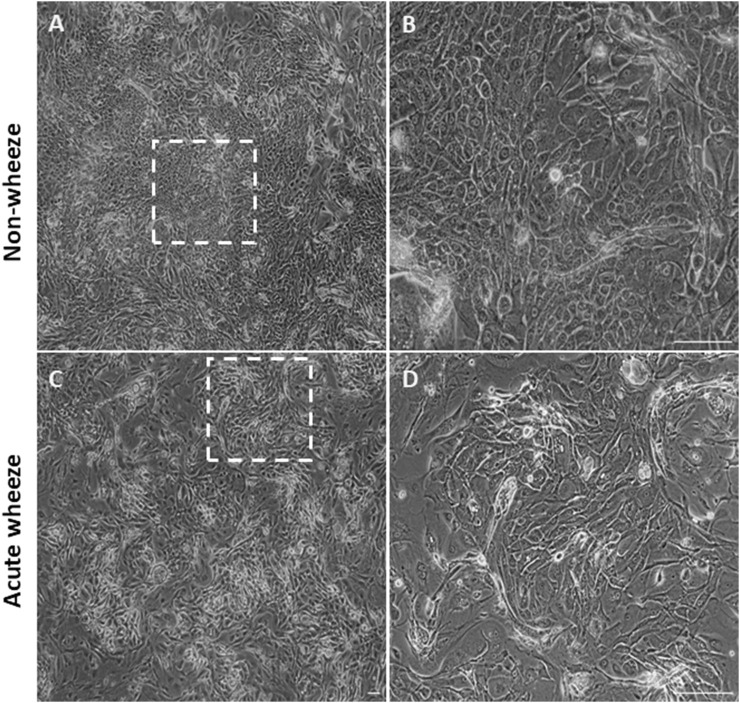
Epithelial morphology between non-wheeze and acute wheeze cultures. **(A,B)** Non-wheeze AEC monolayer at confluence, post thaw, showing characteristic polygonal cobblestone morphology, as highlighted by the dotted box. **(C,D)** Acute wheeze AEC monolayer at confluence, post thaw. These cultures demonstrated heterogeneity in cell morphology, a key biological finding of the study. This observation is attributed to a mix of cellular morphologies, ranging from typical epithelial cobblestone to mesenchymal-like areas, as highlighted by the dotted box. Representative brightfield image of n = 7 (Non-wheeze) and n = 11 (Acute wheeze) at ×4 objective **(A,C)** and ×40 objective **(B,D)**. Scale bar = 100 µm.

Cryopreserved upper AECs of both acute wheeze and non-wheeze cohorts were thawed, cell counts performed, and viability assessed via trypan blue exclusion. Mean cell viability at thaw (Figure 1A) for the acute wheeze cohort was 54% ± 24% with an average live cell yield (Figure 1B) of 3.81 ± 2.18 × 10^5^ cells/mL and was not significantly different from the non-wheeze cohort, 63% ± 12% with an average live cell yield of 3.73 ± 1.25 × 10^5^ cells/mL. At confluence, the mean cell viability at first passage (Figure 1C) was 93% ± 3%, with an average live cell yield (Figure 1D) of 12.17 ± 4.99 × 10^5^ cells/mL for the acute wheeze cohort. This was not significantly different to the non-wheeze cohort, 91% ± 11% with an average live cell yield of 11.26 ± 1.25 × 10^5^ cells/mL. Acute wheeze cohorts took 9.6 ± 3.3 days from first thaw to reach confluence, and this was not significantly different to the 9.86 ± 2.5 days taken by the non-wheeze cohort (Figure 1E). Acute wheeze cohort had a 91% success rate of cell culture establishment, with 11 of the 13 samples successfully establishing compared to 62% success rate, with 8 of the 13 samples successfully establishing for the non-wheeze cohort. Both non-wheeze (Figures 2A,B) and acute wheeze (Figures 2C,D) cohorts had similar cell densities and demonstrated typical polygonal cobblestone pattern characteristic of epithelial cells, however, the acute wheeze cohort exhibited a wide range of varying morphology, from epithelial-like cobblestone colonies to elongated morphology typical of mesenchymal-like cells (Figures 2C,D).

### 3.2 Epithelial cell lineage shifts towards a more differentiated state in acute wheeze

**FIGURE 3:**
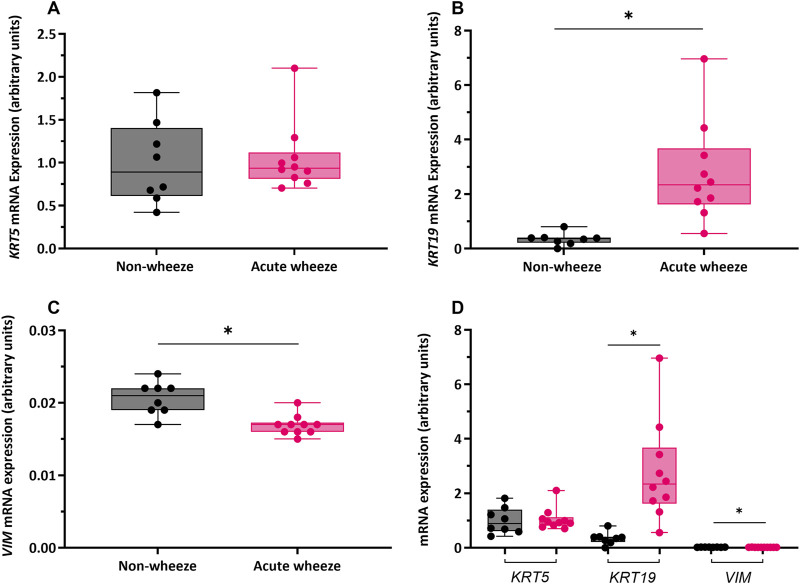
Gene expression of epithelial lineage markers cytokeratin (*KRT)-5, -19* and mesenchymal lineage marker vimentin (*VIM*) in non-wheeze and acute wheeze cultures. **(A)** *KRT5* mRNA expression between non-wheeze (n = 8) and acute wheeze (n = 10) cultures were not significantly different. **(B)** *KRT19* mRNA expression was significantly higher in acute wheeze (2.7 ± 1.8 arbitrary units (AU), n = 10) compared to non-wheeze (0.3 ± 0.2AU, n = 8) cultures. **(C)** *VIM* mRNA expression was significantly lower in acute wheeze (0.01 ± 0.001AU, n = 10) cultures in contrast to non-wheeze (0.02 ± 0.002AU, n = 8) cultures. **(D)** Overview comparison of epithelial and mesenchymal lineage mRNA markers between non-wheeze and acute wheeze cultures. Acute wheeze cultures showed significantly greater expression of *KRT19*. 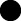
Non-wheeze, 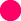
Acute wheeze. AU represents gene-specific relative quantification and are not for direct comparison of absolute expression levels between different genes. *p < 0.05.

RT-qPCR with targeted primers (Table 1) was used to assess gene expression of the epithelial lineage markers cytokeratin 5 and 19 (*KRT5* and *KRT19*), and the mesenchymal lineage marker vimentin (*VIM*) as markers of cell lineage and differentiation. Expression of *KRT5* was not significantly different between the acute wheeze (1.05 ± 0.4 arbitrary unit (AU), Figure 3A) and non-wheeze (0.99 ± 0.4AU, Figure 3A) cohorts. However, *KRT19* expression was significantly higher in acute wheeze (2.7 ± 1.8AU, Figure 3B), compared to controls (0.3 ± 0.2AU, Figure 3B). A significantly lower *VIM* expression was observed in the acute wheeze cohort (0.01 ± 0.001AU, Figure 3C) in contrast to controls (0.02 ± 0.002AU; Figure 3C). Overall, both cohorts showed similar high expression of the epithelial lineage markers, *KRT5*, followed by *KRT19*, which was significantly greater in the acute wheeze cohort. Expression levels of the mesenchymal lineage marker, vimentin, was low, in both cohorts, although this was significantly lower in the acute wheeze cohort (Figure 3D).

**FIGURE 4:**
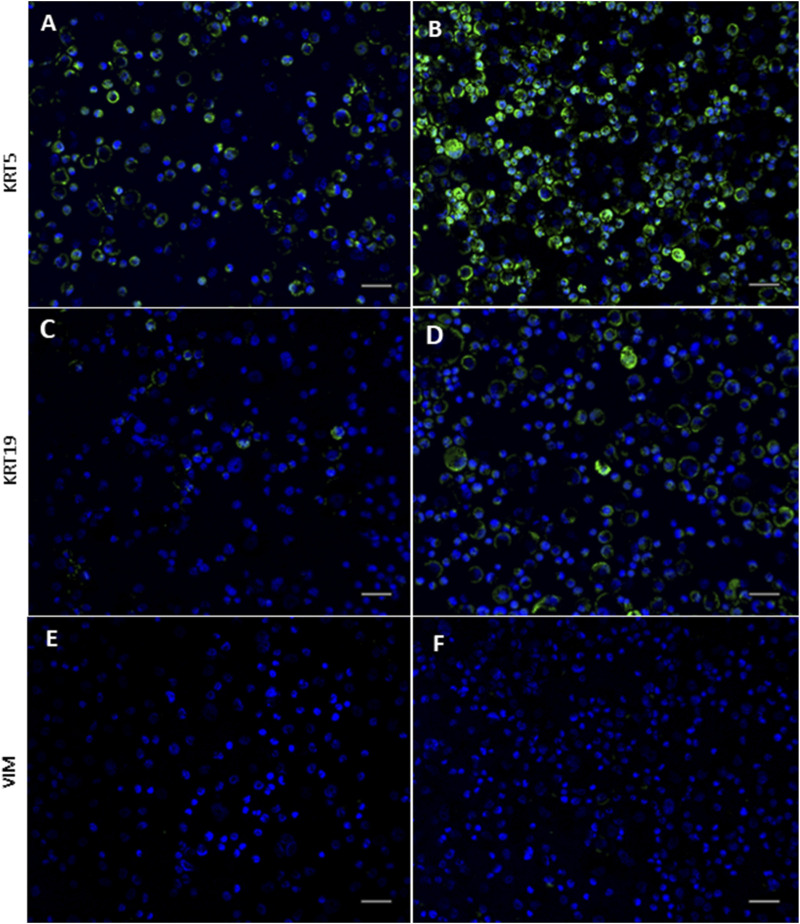
Expression of epithelial and mesenchymal lineage markers in non-wheeze and acute wheeze cells. **(A,B)** Cytokeratin (KRT)-5 staining intensity is markedly stronger in acute wheeze **(B)** compared to non-wheeze **(A)** cells. **(C,D)** Cytokeratin (KRT)-19 staining intensity is also markedly stronger in acute wheeze **(D)** compared to non-wheeze **(C)** cells. **(E,F)** Mesenchymal lineage marker, vimentin staining intensity was not detected in both cohorts. DAPI nuclear stain represented in blue on all images. Representative images of n = 7 study participants per cohort. Scale bar; 100 µm.

Immunocytochemical staining of cytospins demonstrated marked differences in KRT5 protein expression between the cohorts (Figures 4A,B), with greater staining intensity observed within the acute wheeze cohort (Figure 4B). Staining intensity for KRT19 (Figures 4C,D) similarly showed greater intensity for KRT19 protein in the acute wheeze cohort (Figure 4D). The markedly greater staining intensity of both KRT5 and KRT19 from the acute wheeze cohort compared to the non-wheeze cohort is strongly indicative of higher protein abundance. Vimentin, indicative of mesenchymal lineage, was not observed in either cohort (Figures 4E,F).

### 3.3 Comparison of epithelial cell responses between acute wheeze and non-wheeze cohorts

#### 3.3.1 Proliferative capacity is reduced in AECs from children with acute wheeze

**FIGURE 5:**
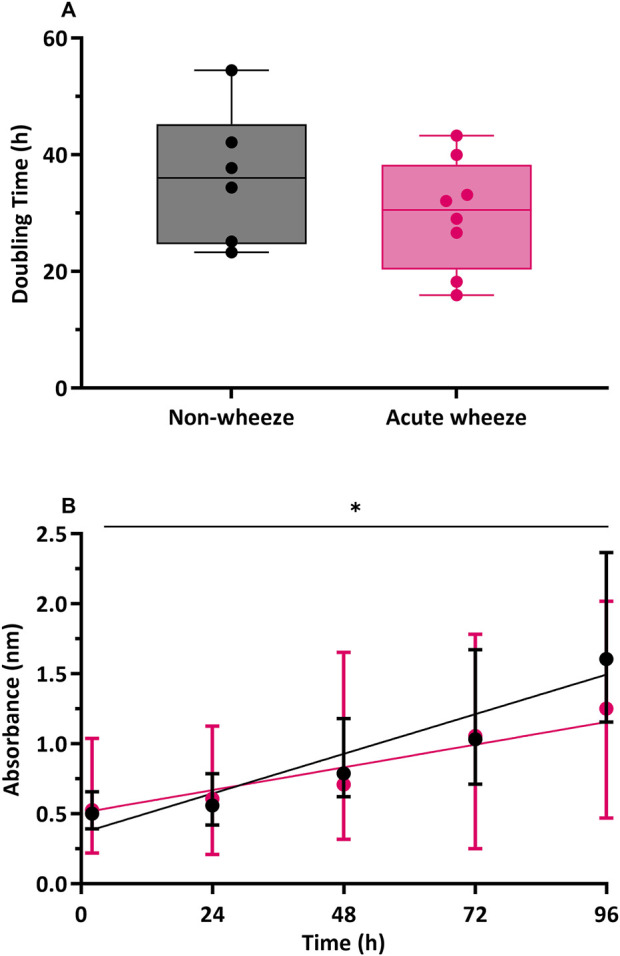
Cell population doubling and proliferation in non-wheeze and acute wheeze cultures. **(A)** Cell populations in non-wheeze cultures occurred over a mean doubling time of 36.2 ± 11.5 h and was not significantly different to cell populations in the acute wheeze cultures, requiring an average of 29.8 ± 8.9 h. **(B)** Cell proliferation in non-wheeze cultures demonstrated a strong relationship between time and proliferation (R^2^ = 0.6645, p < 0.0001), in contrast to a weaker relationship, observed in the acute wheeze cultures (R^2^ = 0.2324, p < 0.007). Proliferative capacity of acute wheeze cultures is significantly slower compared to non-wheeze cultures. 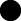
Non-wheeze, 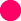
Acute wheeze, *p < 0.05.

Direct cell counts were also used to determine proliferation rates and doubling times. In the absence of exogeneous stimuli, as shown in Figure 5A, epithelial cells from the acute wheeze and non-wheeze cohorts exhibited similar doubling times (29.8 ± 8.9 and 36.2 ± 11.5 h, respectively) and were not significantly different. A moderately strong relationship between time and cell proliferation was observed in the non-wheeze cultures (R^2^ = 0.6645, Figure 5B), in contrast to the acute wheeze cultures, which demonstrated a weaker relationship between time and cell proliferation (R^2^ = 0.2324, Figure 5B). Notably, proliferative capacity of epithelial cells from the acute wheeze cultures were significantly slower (Figure 5B), compared to non-wheeze controls.

#### 3.3.2 Pro-inflammatory IL-1β levels are constitutively higher in acute wheeze

**FIGURE 6:**
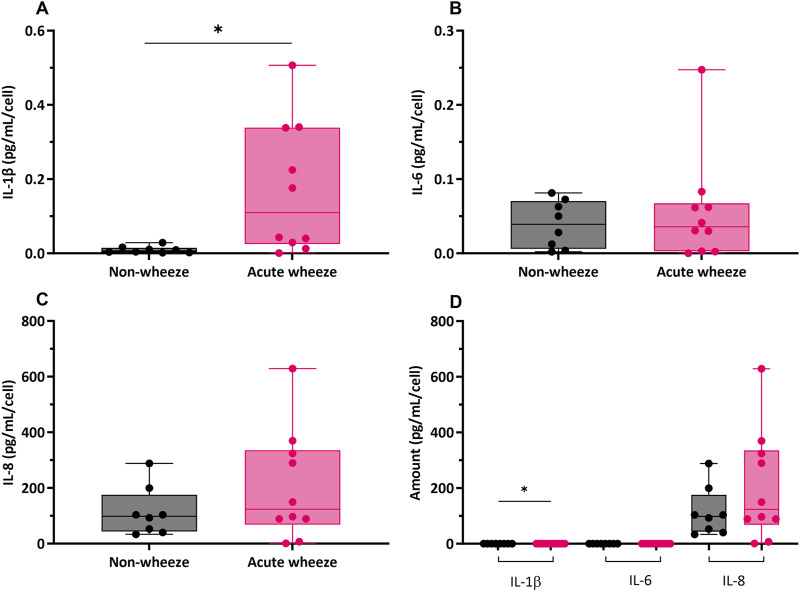
Cytokine mediator expression of interleukin (IL)-1β, −6 and −8 in non-wheeze (n = 8) and acute wheeze (n = 10) cultures. **(A)** IL-1β protein levels were significantly higher in acute wheeze (0.17 ± 0.17 pg/mL/cell; p < 0.05) compared to non-wheeze (0.0095 ± 0.009 pg/mL/cell) cultures. **(B)** IL-6 cytokine protein levels between non-wheeze and acute wheeze cultures were not significantly different. **(C)** IL-8 cytokine protein levels between non-wheeze and acute wheeze cultures were similarly not significantly different. **(D)** Overview comparison of IL-1β, −6 and −8 between non-wheeze and acute wheeze cultures. Acute wheeze cultures showed significantly greater expression of IL-1β, with both cohorts demonstrating similarly high levels of IL-8. 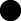
Non-wheeze, 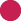
Acute wheeze. *p < 0.05.

The constitutive production of cytokine mediators, including interleukin (IL)-1β, −6 and −8 are shown in Figure 6. Levels of IL-1β expression were significantly higher in the acute wheeze cohort when compared to non-wheeze cohort (0.17 ± 0.17 pg/mL/cell vs. 0.0095 ± 0.009 pg/mL/cell respectively; Figure 6A). However, both IL-6 and IL-8 levels were not significantly different between the acute wheeze and non-wheeze cohorts (Figure 6B, C respectively). Overall, both cohorts demonstrated similar levels of IL-1β and −6 cytokine protein expression followed by IL-8 protein levels (Figure 6D). Inactivated and activated TGF-β levels from both cohorts were below the limits of assay detection (data not shown).

#### 3.3.3 Epithelial barrier integrity is compromised in AECs from children with acute wheeze

**FIGURE 7:**
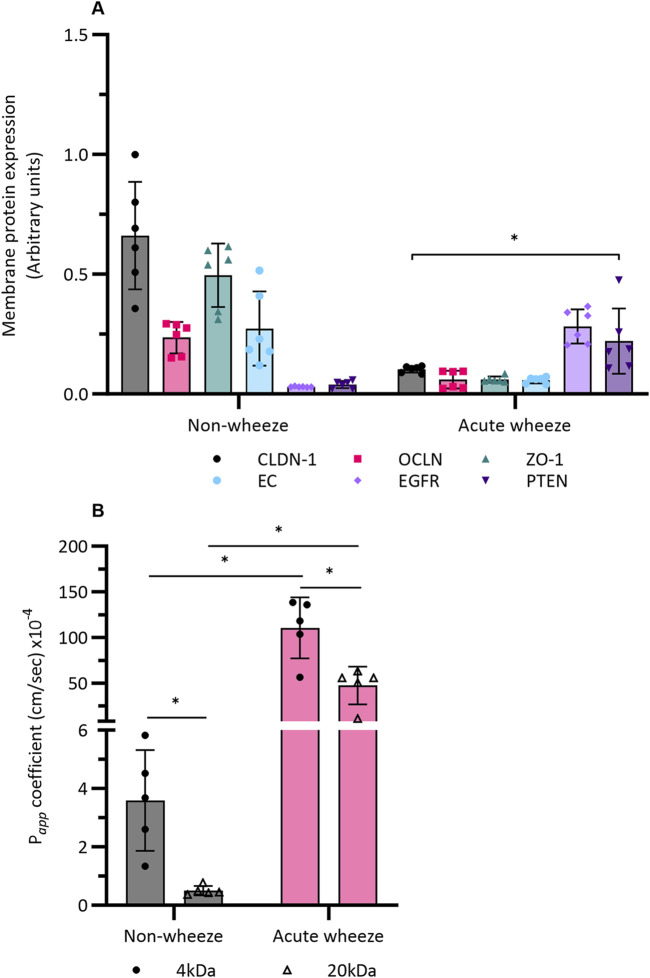
Epithelial integrity and barrier function in non-wheeze (n = 6) and acute wheeze cohort (n = 6). **(A)** Membrane protein expression, quantified via an In-Cell Western^™^ assay, of the TJ and AJ protein claudin-1, occludin, zonula-occluden-1 and e-cadherin were significantly lower in the acute wheeze cohort in contrast to the non-wheeze cohort (CLDN: 0.1 ± 0.01AU vs. 0.6 ± 0.2AU; OCLN: 0.06 ± 0.03AU vs. 0.2 ± 0.06AU; ZO-1: 0.06 ± 0.01AU vs. 0.4 ± 0.1AU; EC: 0.05 ± 0.01AU vs. 0.2 ± 0.1AU). Epidermal growth factor receptor and PTEN protein expression were significantly higher in acute wheeze compared to non-wheeze cohort (EGFR: 0.2 ± 0.07AU vs. 0.02 ± 0.001AU; PTEN: 0.2 ± 0.1AU vs. 0.03 ± 0.01AU). * denotes significance (p < 0.05) of *each* protein target *between* the acute wheeze and non-wheeze cohort. **(B)** Paracellular permeability to small (4-kDa) and large (20-kDa) FITC-dextran were significantly higher in acute wheeze compared to non-wheeze cohort (4-kDa: 110.4 ± 33.4 × 10^−4^ cm/s vs. 3.5 ± 1.7 × 10^−4^ cm/s; 20-kDa: 47.1 ± 20.6 × 10^−4^ cm/s vs. 0.5 ± 0.1 × 10^−4^ cm/s, n = 5). Within the non-wheeze and acute wheeze cohort, paracellular permeability to 4-kDa FITC-dextran was significantly higher compared to 20-kDa (Non-wheeze: 3.5 ± 1.7 × 10^−4^ cm/s vs. 0.5 ± 0.1 × 10^−4^ cm/s; Acute wheeze: 110.4 ± 33.4 × 10^−4^ cm/s vs. 47.1 ± 20.6 × 10^−4^ cm/s). *p < 0.05.

Intrinsic protein expression for tight junctions (TJ), claudin (CLDN)-1, occludin (OCLN), zonula occludens (ZO)-1; adherens junctions (AJ), e-cadherin (EC); epidermal growth factor receptor (EGFR) and the tumor suppressor, phosphatase and tensin homologue (PTEN) were assessed in submerged monolayer cells from both cohorts. Relative to cell numbers, expression of the TJ and AJ proteins, claudin-1, occludin, ZO-1 and e-cadherin were significantly lower in the acute wheeze compared to the non-wheeze cohort (6-, 3-, 8- and 4-fold respectively; Figure 7A). In contrast, both epidermal growth factor receptor and PTEN protein expression were significantly higher in the acute wheeze compared to the non-wheeze cohort (9- and 5-fold respectively; Figure 7A).

Having shown intrinsic protein differences between both cohorts, paracellular permeability across a confluent monolayer of cultured cells from both cohorts was subsequently assessed. In addition to 20 kDa FITC-dextran, a smaller, 4 kDa sized FITC-dextran was also used in the assessment. Intrinsic paracellular permeability to the smaller, 4 kDa FITC-dextran was significantly higher in the acute wheeze compared to the non-wheeze cohort (30-fold; Figure 7B). Paracellular permeability to the larger, 20 kDa FITC-dextran was also significantly greater in the acute wheeze in contrast to non-wheeze cohort (94-fold; Figure 7B) cohort. Within the non-wheeze and acute wheeze cohorts, paracellular permeability to the smaller 4 kDA size FITC-dextran was significantly greater than the larger 20 kDa FITC-dextran (7- and 2-fold respectively, Figure 7B).

## 4 Discussion

This study addressed the hypothesis that the intrinsic epithelial characteristics and barrier integrity of upper AECs from children with acute wheeze is inherently different to those without wheeze. We demonstrate that, upper AECs from children with acute wheeze displayed characteristics of a more differentiated phenotype, evidenced by a significantly greater gene expression of *KRT19*. Interestingly, while there was no significant difference in gene expression of *KRT5*, a markedly stronger KRT5 protein expression via immunocytochemical staining was observed in the acute wheeze cohort, suggestive of potential post-transcriptional regulation. Despite similar population doubling times between the acute wheeze and non-wheeze cohorts, the proliferative capacity of epithelial cells from children with acute wheeze was significantly slower than controls. In addition, epithelial cells from children with acute wheeze constitutively produced substantially higher levels of the pro-inflammatory mediator IL-1β, with similar levels of IL-6 and IL-8 in both cohorts. Furthermore, expression of key epithelial junctional proteins were also significantly lower, which was concomitant with increased paracellular permeability towards different sized FITC-dextran in cultures from children with acute wheeze compared to non-wheeze controls.

The mixed morphological characteristics observed in the acute wheeze cultures, together with a reduced proliferative capacity, supports the concept of an altered epithelial state. The finding of similar *KRT5* gene expression levels between the cohorts suggests that both cell populations are derived from a common basal, progenitor-like cell type. This is consistent with studies of asthmatic epithelium which describe a persistent basal progenitor phenotype (Stefanowicz et al., 2012). However, the greater *KRT19* expression within the acute wheeze cohort indicates a more differentiated phenotype, which could potentially be attributed to the time of sample acquisition, when cellular repair processes are active during an exacerbation (Davis and Wypych, 2021).

We also showed increased mediator release of IL-1β from upper AECs of children with acute wheeze compared to non-wheeze controls, which may indicate that the cells are still influenced by the upper airway environment at the time of acquisition. The maintenance of this elevated IL-1β release following subsequent cell passages could also reflect an inherent difference between the epithelium from the two cohorts. These findings are in agreement with other studies that have reported similar findings in individuals with wheeze or asthma (Pringle et al., 2012; Baines et al., 2017; Evans et al., 2018; Wei et al., 2021). The fact that our primary AECs were obtained at the time of a wheezing exacerbation may explain some apparent discrepancies with other studies where samples were obtained during non-exacerbating periods (Kicic et al., 2006; Turner et al., 2018).

A pivotal role of the epithelium is to provide a selective barrier, which is achieved via the maintenance of intercellular junctions. Impairment of these junctions has been shown to be involved in the progression of airway diseases, including asthma (Heijink et al., 2014; Steelant et al., 2016; Chan et al., 2017). Our observations of lower expression of key junctional proteins, claudin-1, occludin, ZO-1, and e-cadherin, corroborate our earlier findings in children with mild asthma (Looi et al., 2018) and suggest the airway epithelium in children with acute wheeze is inherently impaired. This compromised barrier is further evidenced by the higher paracellular permeability observed in the acute wheeze cohort. Our investigation into key regulatory proteins further highlights a dysregulated state. We observed significantly higher expression of both EGFR and the tumor suppressor, PTEN, in the acute wheeze cohort. Increased EGFR expression is consistent with other reports in asthma and may contribute to airway remodeling if chronically activated (Amishima et al., 1998; Burgel and Nadel, 2004). While abundant PTEN protein is contradictory with its known role as an inhibitor of cellular migration, which is delayed in asthmatic cells (Iosifidis et al., 2020), its activity is subject to complex post-translational regulation (Yamada and Araki, 2001). Collectively, the altered expression of these key regulatory proteins depicts an epithelium that is inherently impaired in its ability to maintain an effective barrier and orchestrate repair, potentially increasing vulnerability to further exacerbations by other stimuli.

It is also important to acknowledge the limitations of this study. The findings are based on a submerged monolayer culture model comprising a homogeneous population of basal cells. While this has allowed us to uncover intrinsic abnormalities in this main epithelial progenitor cell, advancing these assessments in well-differentiated air-liquid interface cultures will offer greater insights into potential disease mechanisms. A crucial consideration is the inherent heterogeneity within the acute wheeze cohort. Early-life wheezing is a broad clinical phenotype, and only a subset of these children will progress to develop persistent asthma. It is possible that the variations observed in our results reflect underlying endotypes, with some children displaying a more aberrant epithelial phenotype that may represent a higher risk for future asthma. The current study’s sample size and cross-sectional design preclude a formal cluster analysis to identify such subgroups. Future longitudinal studies are essential to link these baseline cellular characteristics to long-term clinical trajectories.

Furthermore, our findings are based on a cohort from a single, major pediatric hospital. A single-site study design may limit the generalizability of the findings to other populations with different genetic backgrounds, ancestries, or environmental exposures. Additionally, a significant potential confounding factor is the difference in atopic status between the cohorts, with a significantly higher prevalence of atopy in the acute wheeze group compared to the non-wheeze group. Atopy and allergic rhinitis are known to independently compromise epithelial barrier function. Therefore, some of the observed barrier defects could be attributed to atopy rather than, or, in addition to, the acute wheeze phenotype itself. Disentangling these factors and confirming our observations in diverse populations will require future multi-center studies with cohorts specifically matched for atopic status.

In conclusion, we are the first to successfully demonstrate that an *in vitro* monolayer culture model of upper airway epithelium of children with acute wheeze can be established and maintained, via conditional reprogramming. Our observations indicate that the monolayer culture model is suitable for functional studies such as the interrogation of epithelial lineage, cytokine mediator release and epithelial barrier integrity and function. We also showed that despite similarities between the airway epithelium of children with acute wheeze and non-wheezing children, there exists, at a basal cell level, an innate difference within the acute wheeze cohort, characterized by altered differentiation, slower proliferation, a pro-inflammatory state and most critically, a compromised epithelial barrier.

## Data Availability

The original contributions presented in the study are included in the article/supplementary material, further inquiries can be directed to the corresponding author.

## References

[B27] Amishima M. Munakata M. Nasuhara Y. Sato A. Takahashi T. Homma Y. (1998). Expression of epidermal growth factor and epidermal growth factor receptor immunoreactivity in the asthmatic human airway. Am. J. Respir. Crit. Care Med. 157 (6), 1907–1912. 9620926 10.1164/ajrccm.157.6.9609040

[B1] Baines K. J. Fu J. McDonald V. M. Gibson P. G. (2017). Airway gene expression of IL-1 pathway mediators predicts exacerbation risk in obstructive airway disease. Int. J. chronic Obstr. Pulm. Dis. 12, 541–550. 10.2147/COPD.S119443 PMC5308595 28223794

[B28] Burgel P. Nadel J. (2004). Roles of epidermal growth factor receptor activation in epithelial cell repair and mucin production in airway epithelium. Thorax 59 (11), 992. 15516478 10.1136/thx.2003.018879 PMC1746853

[B2] Chan T. K. Tan W. Peh H. Y. Wong W. (2017). Aeroallergens induce reactive oxygen species production and DNA damage and dampen antioxidant responses in bronchial epithelial cells. J. Immunol. 199 (1), 39–47. 10.4049/jimmunol.1600657 28526682

[B3] Davis J. D. Wypych T. P. (2021). Cellular and functional heterogeneity of the airway epithelium. Mucosal Immunol. 14 (5), 978–990. 10.1038/s41385-020-00370-7 33608655 PMC7893625

[B4] de Boer W. I. Sharma H. S. Baelemans S. M. I. Hoogsteden H. C. Lambrecht B. N. Braunstahl G. J. (2008). Altered expression of epithelial junctional proteins in atopic asthma: possible role in inflammation. Can. J. Physiology Pharmacol. 86 (3), 105–112. 10.1139/y08-004 18418437

[B5] Evans M. D. Esnault S. Denlinger L. C. Jarjour N. N. (2018). Sputum cell IL-1 receptor expression level is a marker of airway neutrophilia and airflow obstruction in asthmatic patients. J. Allergy Clin. Immunol. 142 (2), 415–423. 10.1016/j.jaci.2017.09.035 29103994 PMC6019639

[B6] Fanta C. H. (2009). Drug therapy: asthma. N. Engl. J. Med. 360 (10), 1002–1014. 10.1056/NEJMra0804579 19264689

[B7] Gohy S. Hupin C. Ladjemi M. Z. Hox V. Pilette C. (2020). Key role of the epithelium in chronic upper airways diseases. Clin. and Exp. Allergy 50 (2), 135–146. 10.1111/cea.13539 31746062

[B8] Heijink I. Nawijn M. Hackett T. L. (2014). Airway epithelial barrier function regulates the pathogenesis of allergic asthma. Clin. and Exp. Allergy 44 (5), 620–630. 10.1111/cea.12296 24612268

[B9] Holgate S. T. (2011). The sentinel role of the airway epithelium in asthma pathogenesis. Immunol. Rev. 242 (1), 205–219. 10.1111/j.1600-065X.2011.01030.x 21682747

[B10] Holgate S. T. Wenzel S. Postma D. S. Weiss S. T. Renz H. Sly P. D. (2015). Asthma. Nat. Rev. Dis. Prim. 1 (1), 15025. 10.1038/nrdp.2015.25 27189668 PMC7096989

[B11] Iosifidis T. Sutanto E. N. Buckley A. G. Coleman L. Gill E. E. Lee A. H. (2020). Aberrant cell migration contributes to defective airway epithelial repair in childhood wheeze. JCI Insight 5 (7), e133125. 10.1172/jci.insight.133125 32208383 PMC7205257

[B12] Kenmoe S. Kengne-Nde C. Ebogo-Belobo J. T. Mbaga D. S. Fatawou Modiyinji A. Njouom R. (2020). Systematic review and meta-analysis of the prevalence of common respiratory viruses in children< 2 years with bronchiolitis in the pre-COVID-19 pandemic era. PLoS One 15 (11), e0242302. 10.1371/journal.pone.0242302 33180855 PMC7660462

[B13] Kicic A. de Jong E. Ling K.-M. Nichol K. Anderson D. Wark P. A. (2020). Assessing the unified airway hypothesis in children via transcriptional profiling of the airway epithelium. J. Allergy Clin. Immunol. 145 (6), 1562–1573. 10.1016/j.jaci.2020.02.018 32113981

[B14] Kicic A. Stevens P. T. Sutanto E. N. Kicic-Starcevich E. Ling K. M. Looi K. (2016). Impaired airway epithelial cell responses from children with asthma to rhinoviral infection. Clin. and Exp. Allergy 46 (11), 1441–1455. 10.1111/cea.12767 27238549

[B15] Kicic A. Sutanto E. N. Stevens P. T. Knight D. A. Stick S. M. (2006). Intrinsic biochemical and functional differences in bronchial epithelial cells of children with asthma. Am. J. Respir. Crit. Care Med. 174 (10), 1110–1118. 10.1164/rccm.200603-392OC 16908868

[B16] Looi K. Buckley A. Rigby P. Garratt L. Iosifidis T. Zosky G. (2018). Effects of human rhinovirus on epithelial barrier integrity and function in children with asthma. Clin. and Exp. Allergy 48 (5), 513–524. 10.1111/cea.13097 29350877

[B17] Makrinioti H. Hasegawa K. Lakoumentas J. Xepapadaki P. Tsolia M. Castro‐Rodriguez J. A. (2022). The role of respiratory syncytial virus‐and rhinovirus‐induced bronchiolitis in recurrent wheeze and asthma—A systematic review and meta‐analysis. Pediatr. Allergy Immunol. 33 (3), e13741. 10.1111/pai.13741 35338734

[B18] Martinovich K. Iosifidis T. Ling K. Sutanto E. Kicic-Starcevich E. Looi K. (2015). “Conditionally reprogrammed primary airway epithelial cells successfully maintain lineage, phenotypic and functional characteristics,” in Respirology (Wiley-Blackwell), 70. 10.1038/s41598-017-17952-4 PMC5740081 29269735

[B19] Pringle E. J. Richardson H. B. Miller D. Cornish D. S. Devereux G. S. Walsh G. M. (2012). Nasal and bronchial airway epithelial cell mediator release in children. Pediatr. Pulmonol. 47 (12), 1215–1225. 10.1002/ppul.22672 23024038

[B20] Soriano J. B. Kendrick P. J. Paulson K. R. Gupta V. Abrams E. M. Adedoyin R. A. (2020). Prevalence and attributable health burden of chronic respiratory diseases, 1990-2017: a systematic analysis for the global burden of disease study 2017. Lancet Respir. Med. 8 (6), 585–596. 10.1016/S2213-2600(20)30105-3 32526187 PMC7284317

[B21] Steelant B. Farré R. Wawrzyniak P. Belmans J. Dekimpe E. Vanheel H. (2016). Impaired barrier function in patients with house dust mite–induced allergic rhinitis is accompanied by decreased occludin and zonula occludens-1 expression. J. Allergy Clin. Immunol. 137 (4), 1043–1053.e5. 10.1016/j.jaci.2015.10.050 26846377

[B22] Stefanowicz D. Hackett T.-L. Garmaroudi F. S. Günther O. P. Neumann S. Sutanto E. N. (2012). DNA methylation profiles of airway epithelial cells and PBMCs from healthy, atopic and asthmatic children. PLoS One 7, e44213. 10.1371/journal.pone.0044213 22970180 PMC3435400

[B23] Turner S. Miller D. Walsh G. M. Scaife A. Power U. F. Shields M. D. (2018). Pro‐inflammatory mediator responses from neonatal airway epithelial cells and early childhood wheeze. Pediatr. Pulmonol. 53 (1), 10–16. 10.1002/ppul.23915 29136347

[B24] Wei W. Huang J. Ma Y. Ma X. Fang L. Fang W. (2021). IL‐1 signaling pathway molecules as key markers in childhood asthma. Pediatr. Allergy Immunol. 32 (2), 305–313. 10.1111/pai.13388 33025692

[B25] Wolf S. Perez G. F. Mukharesh L. Isaza N. Preciado D. Freishtat R. J. (2017). Conditional reprogramming of pediatric airway epithelial cells: a new human model to investigate early‐life respiratory disorders. Pediatr. Allergy Immunol. 28 (8), 810–817. 10.1111/pai.12810 28981980 PMC5868353

[B26] Xiao C. Puddicombe S. M. Field S. Haywood J. Broughton-Head V. Puxeddu I. (2011). Defective epithelial barrier function in asthma. J. Allergy Clin. Immunol. 128 (3), 549–556. 10.1016/j.jaci.2011.05.038 21752437

[B29] Yamada K. M. Araki M. (2001). Tumor suppressor PTEN: modulator of cell signaling, growth, migration and apoptosis. J. Cell Sci. 114 (13), 2375–2382. 11559746 10.1242/jcs.114.13.2375

